# Chest Compression With Personal Protective Equipment During Cardiopulmonary Resuscitation: A Randomized Crossover Simulation Study

**DOI:** 10.1097/MD.0000000000003262

**Published:** 2016-04-08

**Authors:** Jie Chen, Kai-Zhi Lu, Bin Yi, Yan Chen

**Affiliations:** From the Department of Anaesthesiology, Southwest Hospital, Third Military Medical University, Chongqing, China.

## Abstract

Following a chemical, biological, radiation, and nuclear incident, prompt cardiopulmonary resuscitation (CPR) procedure is essential for patients who suffer cardiac arrest. But CPR when wearing personal protection equipment (PPE) before decontamination becomes a challenge for healthcare workers (HCW). Although previous studies have assessed the impact of PPE on airway management, there is little research available regarding the quality of chest compression (CC) when wearing PPE.

A present randomized cross-over simulation study was designed to evaluate the effect of PPE on CC performance using mannequins.

The study was set in one university medical center in the China.

Forty anesthesia residents participated in this randomized cross-over study.

Each participant performed 2 min of CC on a manikin with and without PPE, respectively. Participants were randomized into 2 groups that either performed CC with PPE first, followed by a trial without PPE after a 180-min rest, or vice versa.

CPR recording technology was used to objectively quantify the quality of CC. Additionally, participants’ physiological parameters and subjective fatigue score values were recorded.

With the use of PPE, a significant decrease of the percentage of effective compressions (41.3 ± 17.1% with PPE vs 67.5 ± 15.6% without PPE, *P* < 0.001) and the percentage of adequate compressions (67.7 ± 18.9% with PPE vs 80.7 ± 15.5% without PPE, *P* < 0.001) were observed. Furthermore, the increases in heart rate, mean arterial pressure, and subjective fatigue score values were more obvious with the use of PPE (all *P* < 0.01).

We found significant deterioration of CC performance in HCW with the use of a level-C PPE, which may be a disadvantage for enhancing survival of cardiac arrest.

## INTRODUCTION

Following a chemical, biological, radiation, and nuclear (CBRN) incident, the risk of death due to apnea, or cardiac arrest, is very high.^1,2^ Prompt medical care just after CBRN incidents can minimize delay and maximize the chances of survival.^3,4^ As decontamination following a CBRN incident requires a minimum of 12 min per casualty to complete,^4^ most of the time, healthcare workers (HCW) who wear CBRN—personal protection equipment (PPE) have to performed time-critical resuscitation procedures within the warm zone (between contaminated and decontaminated areas) prior to decontamination.^5,6^

The level of PPE is dictated by the nature of the hazardous material and degree of contamination.^7^ At this stage, the specific data on the appropriate level of protection for the HCW performing high-risk procedures, are limited. According to the classification of US Environmental Protection Agency, PPE ranges from A to D, of which level C is commonly used by HCW as a minimum to attend a community emergency response plan.^3,4,6,7^ However, although rigid prerequisites for the protective ability of PPE are obviously essential, it is also important to know whether the equipment impedes HCW during clinical procedures, particularly cardiopulmonary resuscitation (CPR) events such as external chest compression (CC) and endotracheal intubation.^8^

A number of previous studies have assessed the impact of PPE on airway management.^5,8^ However, there is little research available regarding the quality of CC with the use of PPE. In this study, we investigated and evaluated whether CC procedure could be successfully performed while wearing level-C PPE on a manikin. We hypothesized that different protective device (with or without PEE) create differences in performance outcomes, physiological parameters, and fatigue of HCW.

## MATERIALS AND METHODS

### Study Design

This prospective, randomized, crossover design was approval by the Ethics Committee of Southwest Hospital, Third Military Medical University, Chongqing, China (Chairperson: Prof. Jun Wu) and was conducted in a tertiary-referral university hospital in October 13, 2014.

### Participants

A total of 40 anesthesiologists of our hospital with 1 to 4 years of residency experience, who had a basic life support and CPR training course in accordance with the 2010 American Heart Association (AHA) guidelines every year and got a certificate for CPR, were selected based on their availability to participate in the study after signing the written informed consent. Exclusion criteria were muscular skeletal injuries, sprains, pain, heat intolerance, claustrophobia, and pregnant women.

### Study Protocol

Before the study, we explained to the participants that the study compared fatigue and performance of CC when wearing 2 different types of protective devices. The participants were subsequently randomized into 2 groups. By random drawing lots, subjects of odd number were allocated to group 1 and subjects of even number were in the group 2. In group 1, all the participants performed continuous CC without PPE (wearing usual hospital protective clothing of gown and gloves, non-PPE) first and then repeated the same CC wearing level-C PPE. Group 2 performed the test vice versa. The duration of CC was 2 min for each session, alternating with a rest period of 180 min.

A CPR manikin (Resusci Anne Skill Reporter, Laerdal Medical Ltd, Orpington, UK) was located on the floor. All of the participants kneeled beside the manikin's chest to mimic typical resuscitations of bystander. The measurement accuracy of manikin for appropriate hand position, compression rate, compression depth, and chest release was independently verified by investigators through system testing with lower sternum versus other positions, Metronome software (at 80, 100, and 120 beats/min) version 1.1.4 for Apple iOS, ruler-measured depression of manikin chest piston (at 25, 40, and 50 mm), and complete chest release versus incomplete chest release (10-mm compression), respectively. The manikin was connected to a laptop computer for data recording, using the laerdal PC Skill Reporting System (PC Skillmeter, Laerdal Medical, Stavanger, Norway). The CCs data such as rate, depth, number, and the effective compressions were measured to assess the CC performance.

Before and after each CC session, subjective fatigue was evaluated immediately using a 100-point visual analog scale (VAS) ranging from 0 “none” to 100 “extreme exhaustion.” Physiological parameters, including heart rate (HR), blood pressure, and oxygen saturation, were also measured immediately.

Level-C PPE is a complete set of equipment that is currently used by HCW worldwide. The equipment includes safety gloves (Lakeland Neosol Neoprene Gloves, GL-EC30F; Newmarket, Ontario, Canada), chemical protective clothing (Dupont Tyvek Barrier Man “C”, Shanghai, China), a respirator mask (3M Full Facepiece Reusable Respirator 6800, Respiratory Protection, Medium 4/cs, 3M company, 3M center, St Paul, Minnesota) with active filter (3M Gas and Vapour Filter 6099), and safety gumboots (Huili heavy-Duty PVC/Nitrile, WRR0213, Shanghai, China).

### Data Analysis

The primary outcomes were the percentage of effective compressions and the percentage of adequate compressions. An effective compression was identified as 1 CC with a depth of more than 50 mm. The adequate compressions were considered to be compressions with an adequate rate (100–120 per min).

The secondary outcomes included the mean rate and depth of the compressions, the average depth of adequate compressions, and the percentage of effective trials. An effective trial was identified as 1 trial with a mean rate of 100 to 120 per min and more than 80% of compressions deeper than 50 mm.^9^ Additionally, participants’ physiological parameters and VAS of fatigue were evaluated. Aspects such as incomplete release, bad positioning, and excessive compression depth were not included in evaluation.

The sample size was calculated based on the primary outcome. The pilot study showed a 30% difference in the effective compressions rate (the rate of compressions deeper than 50 mm) between the PEE group and the non-PPE group during 2 min of the CC session. Non-PPE group had a higher effective compressions rate than PPE group. Based on an alpha of 0.05 (2-tailed) and a beta error of 0.2, a minimum of 36 participants in each group would be required to allow the detection of a difference between the protective techniques with a power of 80%. In our study, 40 participants were recruited in each group in case there were expulsion cases.

Statistical analysis was performed by SPSS version 17.0 (SPSS, Inc, Chicago, IL). Continuous variables, including CC data, physiological parameters, and VAS, were presented as mean ± standard deviation, whereas categorical variables were expressed as frequency and percentages. Shapiro–Wilk test was used to assess the data distribution. Continuous variables were compared between 2 different device groups by the Wilcoxon signed rank test (non-normal distribution) or paired *t* test (normal distribution), and categorical variables were compared between 2 different device groups by the chi-squared test. A *P* value < 0.05 was set as statistically significant for all analyses.

## RESULTS

### Study Population

**FIGURE 1:**
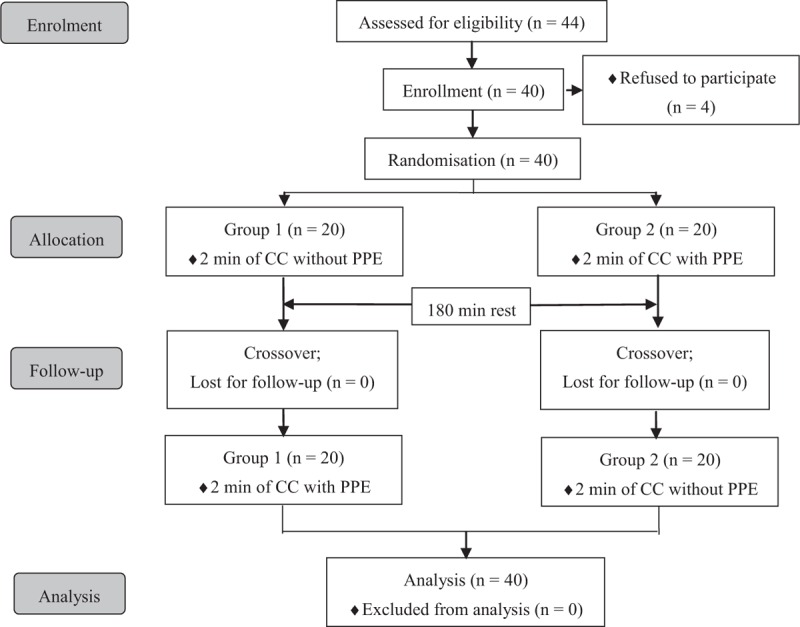
Flow chart of design and recruitment of participants. CC = chest compression, PPE = personal protection equipment.

Forty CPR-certified HCW took part in the study in October 13, 2014. Among them, 20 were women. The mean age of HCW was 27.3 ± 2.6 years, the mean height was 167.0 ± 7.6 cm, and the mean weight was 60.7 ± 12.2 kg (Table 1). The flow chart of this randomized crossover study was shown in Figure 1. None of the participants dropped out from the study.

**TABLE 1:**
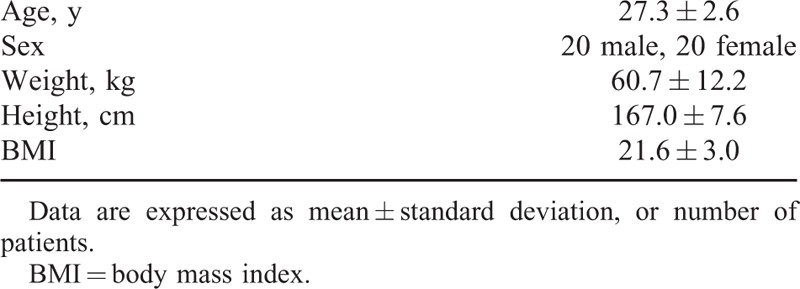
Demographic Characteristics of Participants (n = 40)

### Primary Outcomes

A significant decrease in the percentage of effective compressions with the use of PPE device was observed (41.3 ± 17.1% with PPE vs 67.5 ± 15.6% without PPE, *P* < 0.001; Table 2). Furthermore, there was a significant difference in the percentage of adequate compressions when wearing different protective devices (67.7 ± 18.9% with PPE vs 80.7 ± 15.5% without PPE, *P* < 0.001; Table 2).

**TABLE 2:**
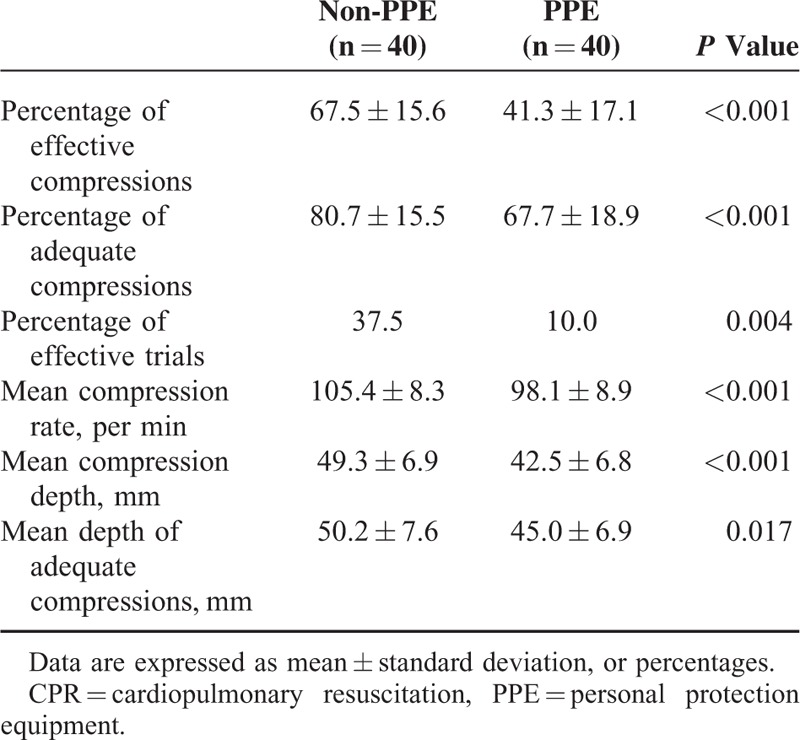
Chest Compression Data During 2 min of CPR Session

### Secondary Outcomes

The mean compression depth and rate were significantly different between trials with and without PPE (all *P* < 0.001; Table 2). Furthermore, we observed that the rate of effective CC trials was 37.5% without PPE in contrast to 10% with PPE (*P* = 0.004; Table 2). The mean depth of adequate compressions was 45.0 ± 6.9 mm with PPE in contrast to 50.2 ± 7.6 mm without PPE (*P* = 0.017; Table 2).

The physiologic variables and perceived exertion of participants measured before and after both sessions of CC were shown in Table 3. There were no significantly differences in the participants’ HR, mean arterial pressure (MAP), oxygen saturation (SpO_2_), and VAS between trials with and without PPE before CC session (*P* = 0.099, *P* = 0.537, *P* = 0.594, and *P* = 0.667, respectively). When comparing post-CC to pre-CC in the same group, the values of HR, MAP, and VAS were significantly higher (all *P* < 0.001), the SpO_2_ value was significantly lower (all *P* < 0.01). After CC session, the values of HR, MAP, and VAS in the PPE group were significantly higher than that in the non-PPE group (*P* = 0.001, *P* = 0.004, and *P* < 0.001, respectively), and the SpO_2_ value between the PPE group and non-PPE group was not significantly different (*P* = 0.943).

**TABLE 3:**
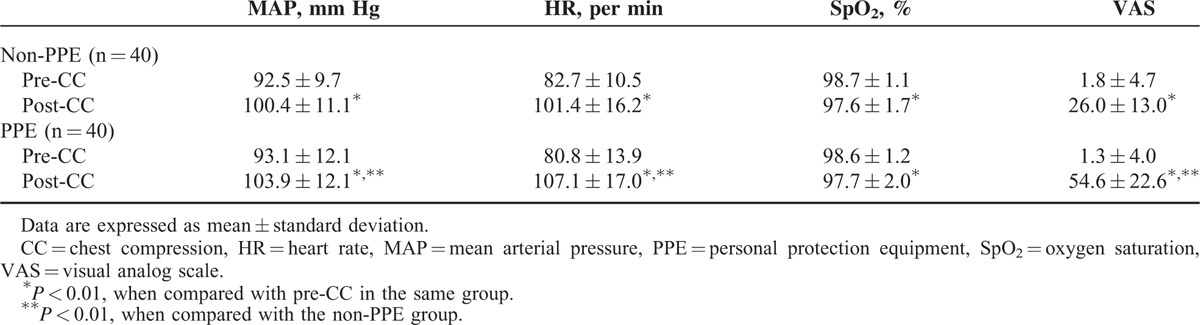
Physiologic Variables and Perceived Exertion Before and After Each Chest Compression Session

## DISCUSSION

To our best knowledge, this is the first study to investigate the influence of PPE on CC by HCW. In this study, we found that wearing level-C PPE in a resuscitation scenario significantly deteriorates the quality of CC and may thus deteriorate outcome and survival.

According to the 2010 CPR guidelines, the importance of serial, rhythmic compressions of the chest during the CPR period has been emphasized over ventilation and drug administration. In addition, in these guidelines, high-quality CC session is defined as a compression depth of at least 51 mm and a rate of at least 100 CCs/min.^10^ The reason for this is that effective CC is essential to provide blood flow during CPR.^11,12^ Our study found the mean rate and depth of CC with the use of PPE went beyond the limits of guideline. However, the mean compression rate and depth are of limited value in evaluating the quality of CC.^9^ It is difficult to be concluded from an average value that whether the rate or depth of a CC was within the target range at any time of the CC trial. For example, almost half of the compressions far above and the other half of the compressions below the target range may still results in an average value within the limits of guideline. Actually the 2010 CPR guidelines have emphasized the requirement for CC that appropriate rate and depth should be obtained simultaneously.^13^ Therefore, in the present study, the percentages of effective compressions as well as the percentages of adequate compressions were utilized as an appropriate index for the evaluation of CC quality.

It is clear that the returning of the spontaneous circulation in patients experiencing cardiac arrest is dependent on the quality of the CPR they receive. However, a number of investigations have demonstrated that rescuers develop immediate fatigue during CPR and the quality of CC declines rapidly after 1 to 3 min of CPR.^14–16^ The 2010 AHA guidelines for CPR, therefore, recommend that rescuers should perform CC and switch roles every 2 min.^10^ In addition, though previous studies that evaluate the performance of medical procedures wearing level-C PPE have found a number of adverse physiological impacts leading to at least a 30% reduction in working ability,^17–19^ the data regarding the acute physiological effects of CC on rescuers wearing level-C PPE are still limited. To achieve the goals of successful CPR and ensuring participants’ safety, according to the 2010 CPR guidelines, therefore, the duration of 2 min of CC was designed in this study protocol which had been approved by our institutional review board. Our study demonstrated a significant deterioration in the overall quality of CPR performance accompanying physiological distress when performing CC with level-C PPE for 2 min. The percentages of effective compressions and adequate compressions with PPE were lower than those without PPE. Moreover, in our study, PPE had no effect on saturation in CC session; but the increases in the HR, MAP, and subjective VAS score values, which may reflect rescuer fatigue, were higher with PPE than that without PPE. Rescuer fatigue, from the rapid depletion of carbohydrate stores, could be the primary cause of deterioration in CC session efficacy.^14,16^ Our study showed that performing CC session when wearing level-C PPE that resulted in a greater workload, is a physical challenge to rescuers. An evaluation of physiologic variables and subjective scales of perceived exertion demonstrated that significant fatigue and inadequate CC are common after 1 min of CPR. This indicates that rescuer fatigue can develop quickly and rescuer performance is lower wearing level-C PEE than that without PPE. Rotating rescuers every 1 min may be, therefore, reasonable to prevent a decrease in compression quality when performing CC session wearing level C PPE.

### Limitations

This study has several limitations. Firstly, this is a manikin study. Although manikins are standardized compared to a great variety of patients, manikin cannot perfectly mimic humans, especially when representing an unconscious, apnea, and pulseless victim. The participants’ attitude toward a simulated CPR may be different from that toward an actual CPR. In our study, participants only focus on adequate compressions, but in real-life conditions they may be distracted by other important interventions (e.g., intubation, defibrillation).^9^ In addition, although the use of simulated cardiac arrest to evaluate CC quality has been well established in the literatures,^9,14,16,18^ it is still debated whether the findings in mannequins are applicable to clinical practice. Observations on compression depth suggest that HCW in reality generally compress too shallowly but in simulated cardiac arrest situation tend to compress too deep.^20^ Secondly, a long PPE wearing time can cause delay in real-life situations and stress discomfort in disaster situations. This factor was not considered in the present study. Lastly, as CPR was conducted for a short period of 2 min only, we cannot extrapolate this finding to the effect of PPE on the quality of CPR when the time is longer than 2 min. Further investigation especially clinical trials are needed for evaluation.

## CONCLUSION

We found significant deterioration of CC performance in HCW when wearing level-C PPE, which may be a disadvantage for enhancing survival of cardiac arrest. The percentages of effective compressions as well as the percentages of adequate compressions were significantly decreased with the use of a level-C PPE. More research is needed to evaluate and modify the CPR strategy when wearing level-C PPE.
